# Integrating Multiple Methods to Validate Key Genes Driving the Progression of Breast Ductal Carcinoma In Situ

**DOI:** 10.3390/cimb47100864

**Published:** 2025-10-20

**Authors:** Minjie Zhong, Shengkai Zheng, Yahui Wen, Juansi Zhang, Jiahui Zhang, Hanwei Wang, Caiqin Mo, Sunwang Xu, Xiangjin Chen

**Affiliations:** 1 Department of Thyroid and Breast Surgery, The First Affiliated Hospital of Fujian Medical University, Fuzhou 350005, China; fjykdxzmj@126.com (M.Z.); zshengki@163.com (S.Z.); wenyahui1214@163.com (Y.W.); zhangjunsi2023@163.com (J.Z.); zjh13972342995@163.com (J.Z.); 13514066453@163.com (H.W.); mocaiqin1990@fjmu.edu.cn (C.M.); 2 National Regional Medical Center, Department of Thyroid and Breast Surgery, Binhai Campus of the First Affiliated Hospital, Fujian Medical University, Fuzhou 350212, China; 3 Fujian Provincial Key Laboratory, Precision Medicine for Cancer, Fuzhou 350000, China

**Keywords:** DCIS infiltrative progression, tumor stroma, tumor microenvironment

## Abstract

Background: Ductal carcinoma in situ (DCIS) is a precursor to breast cancer. The mechanisms by which the stroma of DCIS affects disease progression remain elusive. Thus, the aim of this study is to identify key stroma genes that affect DCIS progression and to define high-risk DCIS cases. Method: Gene expression matrix files from the Gene Expression Omnibus (GEO) database were selected to identify candidate genes associated with the stromal transition from DCIS to invasive ductal carcinoma (IDC). An integrative approach was employed to identify and functionally characterize driver genes of DCIS progression. In vitro experiments were performed to validate the role of these genes. Results: We identified 13 differentially expressed genes (DEGs), of which 5 were selected as candidate drivers. Gene set enrichment analysis (GSEA) revealed the biological functions of RAMP2 and ADM2, while in vitro functional assays demonstrated that ADM2 knockdown and RAMP2 overexpression in breast cancer cell lines significantly suppressed cellular proliferation and invasion. Conclusion: This study identified and validated the roles and functions of ADM2 and RAMP2 and revealed their function as key driver genes in the progression of ductal carcinoma in situ (DCIS). Collectively, our findings elucidate critical genetic mechanisms underlying DCIS progression and provide novel insights for the development of personalized therapeutic strategies.

## 1. Introduction

Ductal carcinoma in situ (DCIS) is a non-obligate precursor to invasive ductal carcinoma (IDC), with its primary clinical challenge being its potential for progression and long-term recurrence. Currently, accurate methods for classifying the risk level of DCIS are lacking [1].

The tumor stroma is a critical mediator in cancer initiation and progression, and targeting the tumor microenvironment has proven effective across various cancers [2,3,4]. During the progression of DCIS towards IDC, the tumor stroma (mesenchymal cells, immune cells, and the extracellular matrix) plays a distinct role, ultimately leading to DCIS lesions having a high risk of progression to IDC. The proportion of CD138+ immune cells is higher in patients with high-risk DCIS [5]. Other studies have also concluded that DCIS lesions with PDGFR α (low)/PDGFR β (high) fibroblasts are more prone to tumor recurrence. Moreover, various molecules and signaling pathways have been identified to play a decisive role in tumor progression [6,7].

**Figure 1:**
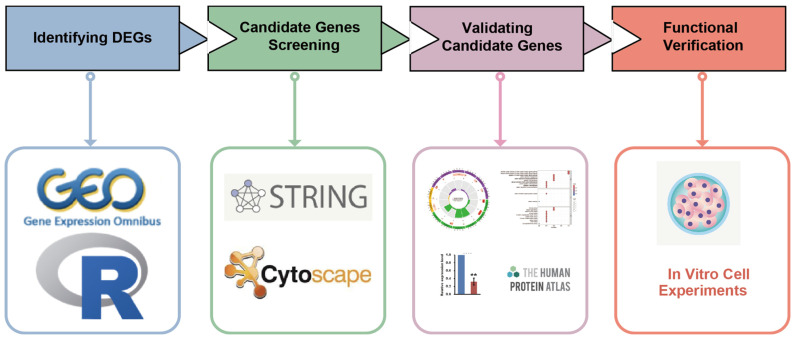
Illustrates the overall experimental design (** *p* < 0.01).

DCIS is primarily detected by mammographic screening, with approximately 75% of cases presenting as microcalcifications [8]. While mammography serves as the key detection tool, for a definitive diagnosis, a breast biopsy is required, which remains the gold standard and is essential for identifying high-risk patients. To build upon this diagnostic pathway, there is a pressing need to elucidate the molecular mechanisms driving DCIS progression to discover biomarkers and targeted therapies. To identify key stromal driver genes and address this gap, we employed an integrative strategy (Figure 1).

In this study, datasets were analyzed, including data on stroma gene expression of DCIS and IDC, to identify differentially expressed genes (DEGs). The PPI network yielded a total of eight genes (ADM2, CALCR, CALCB, CALCA, RAMP2, IAPP, ADM, and RAMP1). Following this, we validated their expression levels in the dataset, which ultimately identified five highly expressed driver genes (ADM2, CALCR, CALCB, RAMP2, and ADM), with ADM2 and RAMP2 showing significant differences in expression levels. ADM2 demonstrated an increased risk for the progression of DCIS, whereas RAMP2 displayed the opposite trend. After constructing siRNAs targeting ADM2 and RAMP2 overexpression plasmids, our results revealed that downregulating ADM2 expression significantly inhibited the proliferation and infiltration of breast cancer cells. Similarly, overexpression of RAMP2 led to a consistent inhibition of both proliferative and invasive capacities, indicating that ADM2 and RAMP2 play a critical role in DCIS progression. Targeting ADM2 and RAMP2 holds potential as a therapeutic strategy to inhibit DCIS progression.

## 2. Materials and Methods

### 2.1. Identification of DEGs

A systematic search was conducted for datasets containing stroma gene expression data on DCIS and IDC from the Gene Expression Omnibus (https://www.ncbi.nlm.nih.gov/geo/) (accessed on 10 September 2024) database. Inclusion Criteria: Transcriptomic datasets containing stromal lesions of both ductal carcinoma in situ (DCIS) and invasive ductal carcinoma (IDC) were included. Exclusion Criteria: Datasets focusing on other disease types, or where patients presented with significant comorbidities, were excluded to ensure a homogeneous cohort for analysis. The “SVA” R package (Version: 3.18) was used to normalize data expression, while the “PCA” R package (Version: 2.10) was used to visualize the normalized data. The “limma” package (Version: 3.8) was used for data analysis and identifying differentially expressed genes using the screening criteria of *p*-value < 0.05 and |log (FC)| > 0.585.

### 2.2. Search Tool for the Retrieval of Interacting Genes (https://cn.string-db.org/)

STRING was used to predict protein–protein interactions among the obtained DEGs and establish a protein–protein interaction (PPI) network (accessed on 15 September 2024). Next, the results were visualized using Cytoscape (Version: 3.5.1) software. Two calculation methods (DMNC and MCC) derived from “cytoHubba” were used to identify critical genes.

### 2.3. Gene Expression Level Validation

The “ggpubr” R package (Version: 0.4) was utilized to visualize the expression of each genes in the retrieved dataset. The “circle” R package (Version: 0.5) was used to display the positions of the expressed genes on the chromosome.

### 2.4. Bioinformatic Validation and Functional Annotation of Driver Genes

Gene Ontology (GO) and Kyoto Encyclopedia of Genes and Genomes (KEGG) analyses facilitate the prediction of potential gene functions. The “clusterProfiler” R package (Version: 4.0) was employed to determine the function of genes and perform GSEA enrichment analysis on individual genes.

CIBERSORT is an R package designed by Newman AM et al. for calculating the level of immune cell infiltration [9]. The annotation file (LM22 signature matrix) was acquired from CIBERSORT (https://cibersortx.stanford.edu/; (accessed on 27 September 2024) and used to calculate differences in the abundance of immune cells between DCIS and IDC. Finally, the “linkET” R package (Version: 0.0.3) was used to visualize the effect of each genes on immune cell abundance.

Kaplan–Meier plotter (https://kmplot.com/analysis/; (accessed on 30 September 2024)) is an online analysis website used for analyzing overall survival rates [10]. The expression of each genes in breast cancer samples, as well as overall survival rate and disease-free survival rate, were assessed. Additional analyses and validation were performed using this GEPIA (accessed on 30 September 2024) to further investigate the identified driver genes [11].

### 2.5. Cell Culture and siRNA Transfection

All cell lines used in this study were authenticated and regularly screened to confirm the absence of mycoplasma contamination. The breast cancer cell line BT-549 (TCHu93, the Cell Bank of Type Culture Collection, CCTCC, Shanghai, China) and MDA-MB-231 (TCHu227, the Cell Bank of Type Culture Collection, CCTCC, Shanghai) were cultured in DMEM medium (Cat. No. MA0212, MeilunBio, Dalian, China) and supplemented with 100 µg/mL streptomycin, 100 U/mL penicillin, and 10% fetal bovine serum (FBS) (Cat. No. A5256701, Gibco, Grand Island, NY, USA) at a temperature of 37 °C and a humidified atmosphere of 5% CO_2_. Small interfering RNAs (siRNA) and non-specific control siRNAs (siNC) used in this study were designed and synthesized by ShangYa (Linjiang, China). Cells were transfected with siRNA using Lipofectamine RNAiMAX Reagent (Cat. No. 13778030, Invitrogen, Waltham, MA, USA). The sense sequence of siRNAs was: siADM2: 5′-CUGUGGUCUGGAAGCUUCATT-3′.

For the RAMP2 overexpression plasmids, the full-length open reading frame (ORF) of human RAMP2 (accession number NM_005854.3, corresponding to the probe 205779_at) was cloned into the pCDH-CMV-MCS-EF1-Puro vector. To generate and infect lentiviruses, HEK293T cells (Cat. No. ACS-4500, American Type Culture Collection, Manassas, VA, USA) at 80–90% confluency were cotransfected with 1.76 μg of knockdown or overexpression plasmid, 1.32 μg of psPAX, and 0.88 μg of pMD2.G in 6 mm dishes with 12 μL of polyethylenimine. After transfection for 6 h at 37 °C, the medium was replaced, and the lentivirus-containing medium was harvested 72 h later. Then, the BC cells were infected by the lentivirus supplemented with polybrene for 24 h, and the puromycin-resistant cells were harvested as the stable transfected cell lines.

### 2.6. Migration and Invasion Assays

In the migration assay, the BT-549 and MDA-MB-231 cells were sequentially starved in the absence of FBS for 4 h, resuspended at a concentration of 2 × 10^4^ cells in 100 μL culture medium without FBS, and seeded in the upper chamber of non-coated Transwell inserts placed in a 24-well plate (Cat. No. 702001, NEST, Wuxi, China). 600 μL culture medium with 10% FBS was subsequently added in the lower chamber and used as a chemoattractant to induce cell migration. After 24 h incubation at 37 °C, the cells were stained with 0.1% crystal violet, and non-migrated or non-invaded cells were gently discarded using a cotton swab and photographed.

### 2.7. Cell Proliferation Assay

Cell proliferation was assessed using the Cell Counting Kit-8 (CCK-8) (Yeasen) method. After 48 h of transfection, BT-549 cells (5000) and MDA-MB-231 cells (1000) were seeded into 96 well plates (Cat. No. 701001, NEST), each containing 100 μL of culture medium. Next, 10 μL of CCK-8 solution was added to the cells at the pre-defined time points, followed by incubation for 1 h at 37 °C. The absorbance of the reaction product was quantified at 450 nm using a Synergy 2 microplate reader (Biotek, Shoreline, WA, USA). For colony formation assay, BT-549 cells (1000) and MDA-MB-231 cells (500) were seeded into a 6-well plate (Cat. No. 703001, NEST). The cells were cultured for 7–14 days. Next, the cells were fixed with 1% paraformaldehyde (Cat. No. G1101-25L, Servicebio, Wuhan, China) for 15 min and stained with crystal violet (Cat. No. GC307002-25g, Servicebio, Wuhan, China) for 15 h.

### 2.8. RNA Extraction and Real-Time Quantitative PCR

Total RNA was extracted from cells using TRI Reagent (Cat. No.T9424, Sigma-Aldrich, St. Louis, MO, USA) following the manufacturer’s protocol. 1 μg of total RNA was reverse transcribed into cDNA using the 1st Strand cDNA Synthesis SuperMix (Cat. No. 11156ES, Yeasen, Shanghai, China). Real-time quantitative PCR (RT-qPCR) was performed in triplicate on an Applied Biosystems ViiA 7 Real-Time PCR system (Applied Biosystem, Waltham, MA, USA). Relative mRNA expression was calculated using the 2−ΔΔCt method and normalized to the internal reference gene ACTB. The primers for real-time PCR are as follows: ADM2, forward 5′-TCCTGCAGCAACAATACCTGC-3′, reverse 5′-GGGTGTTTAGGCTCCTTGACG-3′. RAMP2, forward 5′-AGCCCCTCCGAGGAAGCGGCGCG-3′, reverse 5′-CGGGCCGCCGGCGCGCTCCACCCG-3′.

### 2.9. Statistical Analysis

An unpaired Student’s *t*-test and Chi-square test were used for group comparison of normally distributed measurement data and categorical data, respectively. *p* < 0.05 was considered statistically significant. Statistical significance is indicated as follows: * *p* < 0.05; ** *p* < 0.01; *** *p* < 0.001; or ns, not significant.

## 3. Results

### 3.1. Identification of Candidate Genes for DCIS

**Figure 2:**
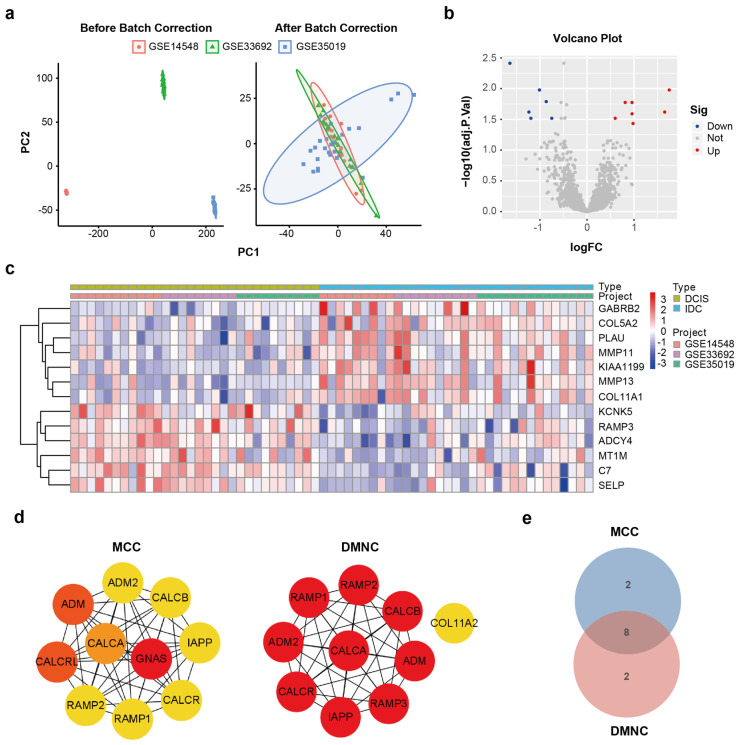
(**a**) Standardized and merged data. (**b**) Volcano and heat maps illustrating the expression levels of DEGs. (**c**) The heat maps show the expression of DEGs. (**d**) MCC and DMNC displaying driver genes. (**e**) The Venn diagram displays intersecting driver genes.

Three microarray profiles containing expression data on DCIS and IDC lesions were downloaded (GSE35019, GSE33692, and GSE14548; Supplemental Tables S1 and S2) and merged (Figure 2a), which led to the identification of 13 DEGs (C7, MMP13, PAMP3, KCNK5, GABRB2, PLAU, SELP, COL11A1, KIAA1199, MT1M, ADCY4, COL5A2, and MMP11) (Figure 2b,c; Supplemental Table S3). Notably, C7, MMP13, and PLAU have been reported as promoters of breast cancer progression [12,13,14].

In order to further examine the role of the DEGs, GO function and KEGG pathway enrichment analyses were carried out (Supplemental Tables S4 and S5). Through GO enrichment analysis, it was determined that DEGs were chiefly enriched in extracellular processes, including extracellular matrix organization, extracellular structure organization, external encapsulation structure organization, and collagen fiber organization, implying an increase in tumor extracellular matrix activity during the progression of DCIS lesions. Enhanced serine metabolism has been shown to be associated with the progression of various cancers [15,16]. The increased metabolic activity of serine identified in this study suggests that serine metabolism may also contribute to the progression of DCIS (Figure S1). KEGG pathway analysis demonstrated that the DEGs were significantly enriched in protein digestion and the absorption pathway (Figure S1), indicating that metabolic enhancement occurs in the early stages of DCIS progression to adapt to abnormal tumor proliferation.

In order to identify driver genes related to the interaction with DEGs, we used the STRING database to construct a protein–protein interaction (PPI) network (Figure S2; Supplemental Table S6). By integrating the results of this PPI analysis with another method (Figure 2d), we identified eight candidate genes (ADM2, CALCR, CALCB, CALCA, RAMP2, IAPP, ADM, and RAMP1) via a VENN diagram (Figure 2e).

### 3.2. Validation of the Expression of Candidate Genes in Breast Cancer

**Figure 3:**
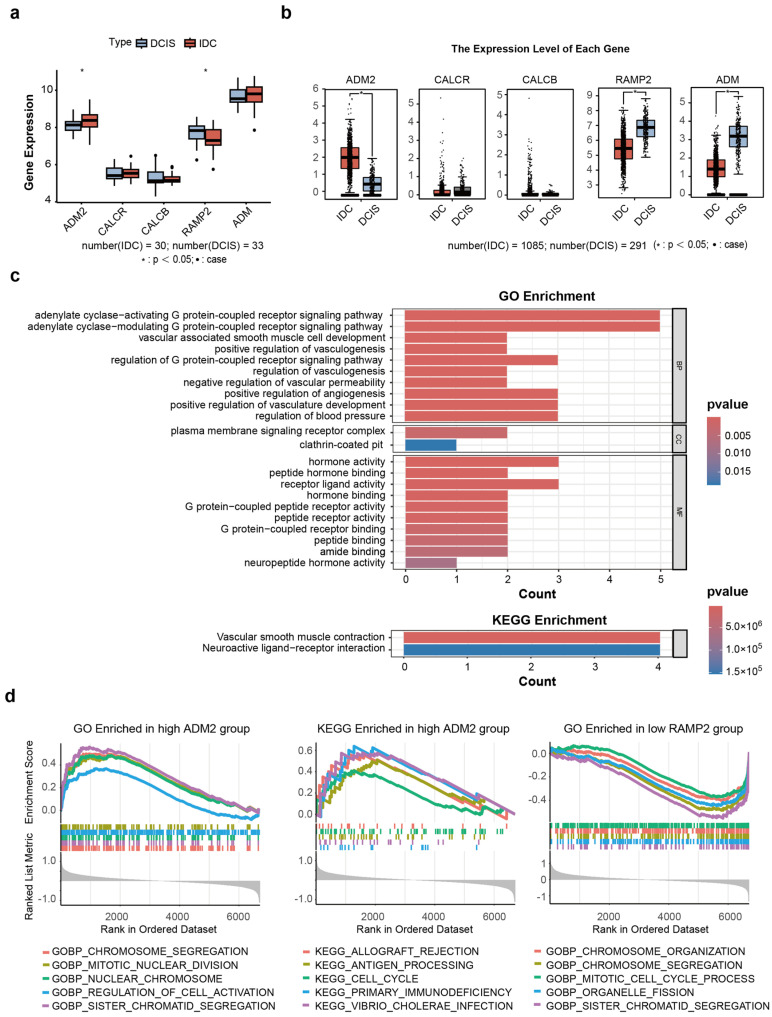
(**a**) Expression profiles of the driver genes in the dataset of this study (* : *p* < 0.05). (**b**) Expression profiles of the driver genes in the TCGA cohort (* : *p* < 0.05). (**c**) Significantly enriched KEGG and GO pathways in driver genes. (**d**) GSEA enrichment analysis of driver genes.

The expression of candidate genes was first validated in three datasets. Notably, data on the expression of only five genes were available in the dataset (ADM; ADM2; CALCR; CALCB; and RAMP2); significant differences were noted in the expression levels of ADM2 and RAMP2 between the DCIS and IDC groups (Figure 3a). Next, the expression of the five genes was verified using GEPIA. With the exception of ADM, the expression trends of the remaining four candidate genes were consistent with our results (Figure 3b). ADM, also referred to as adrenomedullin, is a bioactive peptide that has been associated with a poor prognosis in triple-negative breast cancer patients [17]. It is worth mentioning that the validation data were derived from TCGA, and predominantly comprised transcriptome sequencing results of breast cancer cells from different sources. Our research focused on the tumor matrix, which might account for the inconsistency in ADM expression. However, the consistency in other results suggests that candidate genes can initiate from the matrix level, potentially influencing whether DCIS progresses or not.

### 3.3. Exploring the Effect of Candidate Genes in the Progression of DCIS Lesions

To further investigate the role of driver genes in the progression of DCIS, their position was visualized in chromosomes (Supplementary Figure S2). Our findings unveiled that the candidate genes were significantly enriched in the G protein-coupled receptor signaling pathway, in line with the results of previous research [18,19]. In addition, the candidate genes were related to vascular smooth muscle contraction and neuroactive ligand–receiver interaction (Figure 3c; Supplemental Tables S7 and S8), which suggests that the tumor stroma not only facilitated angiogenesis within the microenvironment but also enhanced intercellular communication, collectively driving the progression of DCIS.

Next, the functions of different expression patterns of candidate genes (Supplementary Figure S3) were examined. Gene set enrichment analysis (GSEA) revealed that ADM2 expression was enriched in GO terms related to chromosomal activity (e.g., chromosome segregation; Supplemental Table S9), while low RAMP2 expression was also associated with various chromosomal processes. Moreover, in KEGG pathway analysis, high ADM2 expression was linked to antigen processing and presentation, suggesting potential roles for both ADM2 and RAMP2 in DCIS progression (Figure 3d). These findings collectively highlight the potential role of immune system dysregulation and increased cell proliferation rates as potential mechanisms underlying tumorigenesis. Subsequently, ADM2 and RAMP2 were identified as “tumor progression-associated genes” (TPAGs), as multiple experimental approaches revealed significant alterations in their expression during DCIS progression.

### 3.4. The Role of Candidate Genes and Immune Cells in the Progression of DCIS

**Figure 4:**
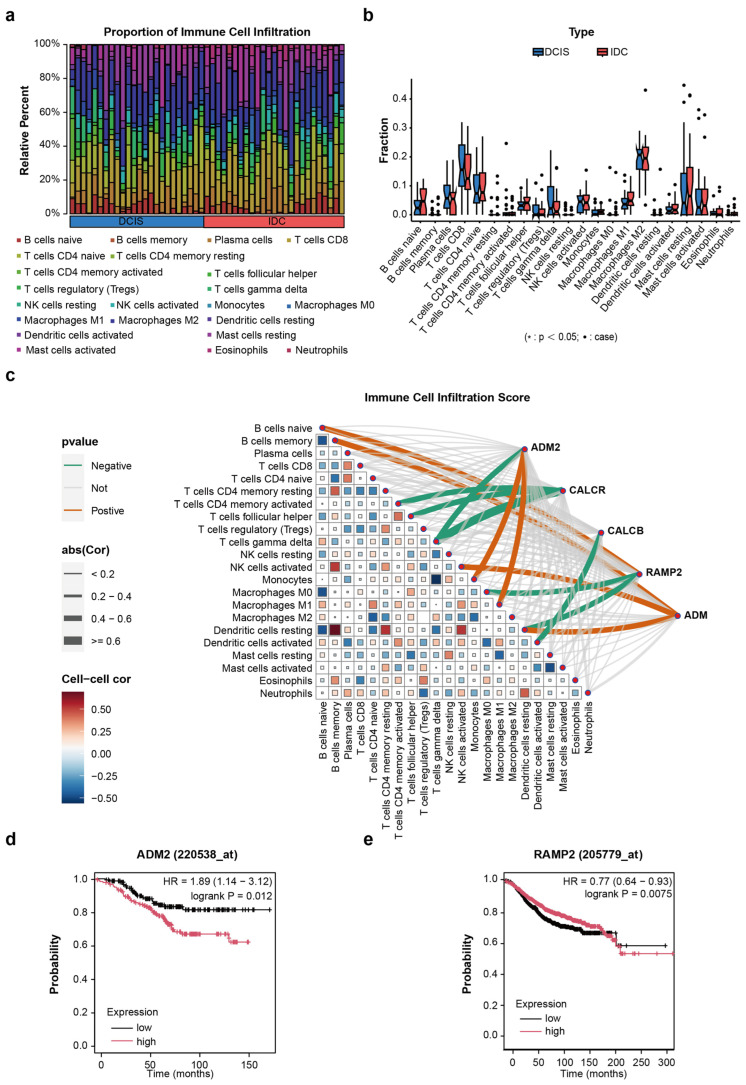
(**a**,**b**) Level of immune cell infiltration. (**c**) Interactions between immune cells and driver genes. (**d**,**e**) Survival curves of TPAGs.

In addition to the role of candidate genes, their effects on immune cells were also examined (Supplemental Table S10). To begin, the infiltration levels of immune cells were visualized in three datasets (Figure 4a,b). Then, the crosstalk between candidate genes and different immune cells was visualized, revealing that ADM2 was negatively associated with gamma delta T cells and positively regulate macrophages and monocytes (Figure 4c). Gamma delta T cells have been reported to exert cytotoxic effects on tumor cells and inhibit their proliferation. On the contrary, monocytes and macrophages can secrete various factors during tumor progression, thereby promoting the infiltration of tumor cells [20,21]. In summary, these findings support a model in which matrix-derived candidate genes may contribute to pro-carcinogenic reprogramming of the immune microenvironment.

### 3.5. The Relationship Between TPAGs and Patient OS

Given that some TPAGs (ADM2 and RAMP2) were differentially expressed, these TPAGs served as the subject for further analysis of the correlation between TPAGs and patient OS (*n* = 4929). As expected, patients with high ADM2 expression exhibited shorter overall survival, whereas high RAMP2 expression was associated with improved survival (Figure 4d,e). Taken together, these findings indicate that ADM2 overexpression and low RAMP2 expression not only play a role in the progression of DCIS towards IDC, but also affect patient prognosis. Therefore, we further evaluated the effects of ADM2 and RAMP2 on the biological functions of breast cancer cell lines in vitro.

### 3.6. Influence of TPAGs on Malignant Behavior in Breast Cancer Cells

**Figure 5:**
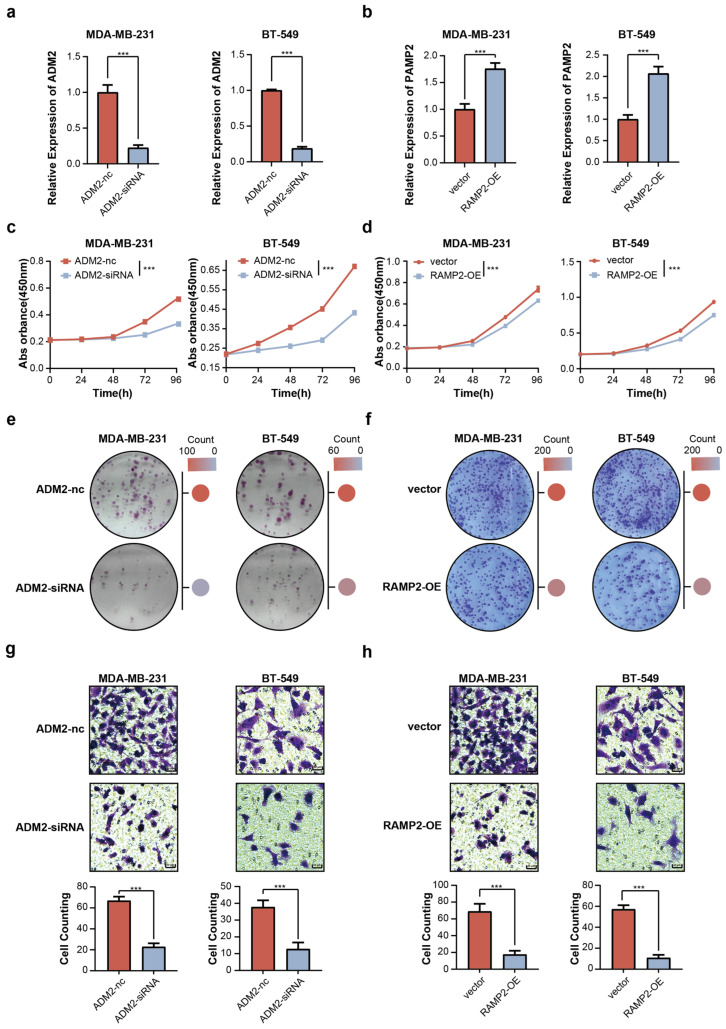
(**a**) RT-qPCR validation of ADM2-siRNA knockdown efficiency (*** : *p* < 0.001). (**b**) RT-qPCR validation of RAMP2 overexpression efficiency. (**c**,**d**) CCK-8 assay shows the proliferation curves after ADM2 knockdown or RAMP2 overexpression in different breast cancer cell lines (*** : *p* < 0.001). (**e**,**f**) Colony formation in different cell lines after knocking down ADM2 and overexpressing RAMP2. (**g**,**h**) Knockdown of ADM2 or overexpression of RAMP2 inhibits cell invasion capacity (*** : *p* < 0.001).

In order to further explore the effect of ADM2 and RAMP2 on breast cancer cells, siRNAs of ADM2 and the overexpression vector for RAMP2 were constructed and transfected into BT-549 and MDA-MB-231 breast cancer cell lines, respectively. RT-qPCR was performed to validate the efficiency of siRNA-mediated knockdown and plasmid-mediated overexpression. The results demonstrate that transfection with siADM2 significantly reduced ADM2 expression, while the RAMP2 wild-type plasmid markedly increased RAMP2 levels in cells (Figure 5a,b). CCK-8 assays demonstrated that ADM2 knockdown significantly suppressed the proliferation of BT-549 and MDA-MB-231 cells, while overexpression of RAMP2 also inhibited proliferation across multiple breast cancer cell lines (Figure 5c,d). Likewise, a colony formation assay yielded similar results, further confirming these phenotypic observations (Figure 5e,f). A hallmark of DCIS progression is the development of microinvasion. Therefore, we further investigated the impact of TPAGs on microinvasive capacity in breast cancer cells. Knocking down ADM2 significantly inhibited the invasive ability of breast cancer cell lines BT-549 and MDA-MB-231. Conversely, overexpression of RAMP2 also reduced the number of invasive breast cells (Figure 5g,h). In summary, our findings demonstrate the functional role of TPAGs in breast cancer progression and support both their potential diagnostic value in predicting the progression of DCIS and the therapeutic potential of targeting TPAGs.

## 4. Discussion

Research shows that 75% of IDC lesions are associated with the initial DCIS lesion, while 18% are unrelated, and the origin of the remaining 7% is unclear [22]. Undoubtedly, the fact that 75% of DCIS cases have the potential to progress to invasive carcinoma is a challenge that should not be overlooked. However, there are currently no molecular markers to predict the progression of DCIS lesions to IDC. While previous studies have assessed the risk of DCIS infiltration through imaging, pathology, and clinical manifestations, their conclusions were not sufficiently reliable. Similar to the diagnosis of breast cancer, which requires confirmation through pathological examination, pathological molecular targets are required for high-risk DCIS lesions to accurately predict disease progression [23,24].

Herein, different datasets were merged to obtain a more comprehensive expression profile of DEGs. Based on our research, DEGs were significantly enriched in serine metabolism-related pathways. Abnormalities in serine metabolism have been confirmed to be associated with the progression of various tumors [25,26,27]. A PPI network was constructed based on the identified DEGs, yielding a total of five potential genes. The expression patterns of these five genes were validated using multiple methodological approaches, and the results were consistent with our initial findings. Of note, all five genes were associated with G protein-coupled receptors (GPCRs), indicating that the activation of GPCR signaling contributes significantly to tumor progression [18,28,29].

The results of other genes were consistent with our results, with the exception of ADM. This discrepancy may be ascribed to the breast cancer data in the TCGA database, as they include data from breast cancer originating from not only ductal cells but also other sources. The findings of our study were primarily derived from the breast stroma, a key component of the tumor microenvironment, whereas the TCGA database largely features transcriptomic data from breast tumor cells. Despite this discrepancy in data sources, the overall expression trends of the driver genes remained consistent, suggesting the presence of a complex signaling crosstalk network between the tumor microenvironment and the progression of ductal carcinoma in situ (DCIS).

Following this, the tumor microenvironments of DCIS and IDC were compared, and no significant difference in immune cell infiltration levels was noted. This result indicates that the tumor microenvironment has already formed during the early stages of disease progression, in line with the results on the parallel occurrence and metastasis of breast cancer [30]. Afterward, the influence of driver genes on different immune cells in different subgroups was evaluated. Importantly, two driver genes (ADM2 and RAMP2, hereafter referred to as TPAGs) with significant expression differences between IDC and DCIS were identified. ADM2 can negatively regulate gamma delta T cell, a T cell subpopulation that inhibits tumor occurrence and development [20,31,32]. In addition, ADM2 can also positively regulate monocytes [33,34] and RAMP2 downregulation can positively regulate B cells [35]. These results suggested a significant correlation between early DCIS progression and the formation of the immune microenvironment. Furthermore, our findings delineate a molecular expression pattern distinct from the conventional markers of ER, PR, and HER2 during DCIS progression. Intriguingly, the identified driver genes, ADM2 and RAMP2, are implicated in calcium metabolism—an observation that may bear a potential link to the frequent detection of DCIS as mammographic calcifications [8,24]. This connection underscores the translational potential that could be uncovered by further investigating these driver genes, positioning them as potential biomarkers for DCIS.

Subsequently, the role of these driver genes in DCIS progression was validated through ADM2 knockdown and RAMP2 overexpression, which, as expected, inhibited the proliferation and migration of breast cancer cell lines. ADM2 is a type of signaling protein generated by the paracrine and autocrine pathways and can regulate downstream signaling by binding to G protein-coupled receptors through different ligands [36]. Based on our results, we speculate that ADM2 can be secreted by tumor stromal cells and bind to G protein-coupled receptors of breast cancer tumor cells through paracrine methods, thereby promoting the proliferation and invasion of breast cancer cells.

Nevertheless, our research also has some limitations that merit acknowledgment. The bioinformatics findings, derived from a cohort of limited sample size, have not been further validated through histological examination of clinical specimens or in in vivo models. Future studies with expanded cohorts and complementary experimental validations are required to confirm the role of these driver genes.

In summary, our study identified TPAGs related to the progression of DCIS to IDC and validated their effects through in vitro experiments, filling a gap in current research in this area. Our research also provides a theoretical basis for the prevention of breast cancer and an effective biological predictive target for the surgical management of DCIS.

## Data Availability

The datasets generated and/or analyzed during the current study are available in the GEO (https://www.ncbi.nlm.nih.gov/geo/) (accessed on 10 September 2024), which contains three datasets (GSE35019, GSE33692, and GSE14548); STRING (https://cn.string-db.org/) (accessed on 15 September 2024); CIBERSORT (https://cibersortx.stanford.edu/) (accessed on 27 September 2024); the GEPIA(http://gepia.cancer-pku.cn/index.html) (accessed on 30 September 2024) repository; and Kaplan–Meier plotter (https://kmplot.com/analysis/); (accessed on 30 September 2024).

## Supplementary Materials

The following supporting information can be downloaded at: https://www.mdpi.com/article/10.3390/cimb47100864/s1.
