# Supplementary material for: Integrating Multiple Methods to Validate Key Genes Driving the Progression of Breast Ductal Carcinoma In Situ
Source: Curr Issues Mol Biol. 2025 Oct 20;47(10):864. doi: 10.3390/cimb47100864 (PMC12564283; doi:10.3390/cimb47100864)
Supplement: Supplementary file 1 [file cimb-47-00864-s001.zip › Supplemental Information.pdf]

Supplemental Information

Integrating Multiple Methods to Validate Key Genes Driving the Progression of Breast Ductal Carcinoma In Situ

Minjie Zhong [1,†], Shengkai Zheng [1,†], Yahui Wen [1], Juansi Zhang [1], Jiahui Zhang [1], Hanwei Wang [1], Caiqin Mo [1], Sunwang Xu [1,2,3,*] and Xiangjin Chen [1,2,*]

1

Department of Thyroid and Breast Surgery, The First Affiliated Hospital of Fujian Medical University, Fuzhou 350005, China; fjykdxzmj@126.com (M.Z.); zshengki@163.com (S.Z.); wenyahui1214@163.com (Y.W.); zhangjunsi2023@163.com (J.Z.); zjh13972342995@163.com (J.Z.); 13514066453@163.com (H.W.); mocaiqin1990@fjmu.edu.cn (C.M.)

2

National Regional Medical Center, Department of Thyroid and Breast Surgery, Binhai Campus of the First Affiliated Hospital, Fujian Medical University, Fuzhou 350212, China

3

Fujian Provincial Key Laboratory, Precision Medicine for Cancer, Fuzhou 350000, China

*Correspondence: xusw1206@163.com (S.X.); rjbhcxj@fjmu.edu.cn (X.C.)

[†]These authors contributed equally to this work.

This supplemental information includes:

Supplemental Figure S1 to S3

Supplemental Table S1 to S10

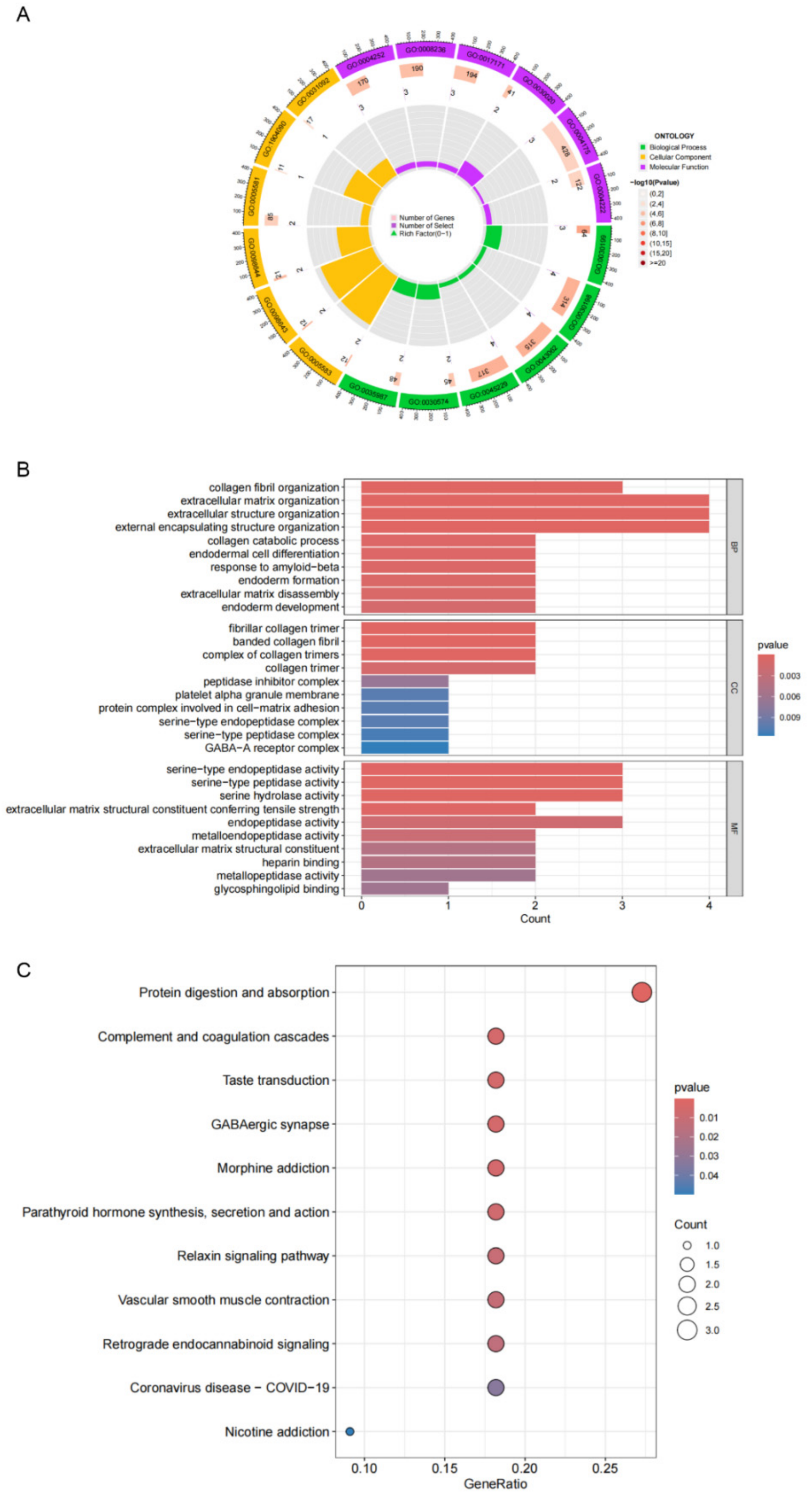

Figure S1. DEGs function. (A) GO functional circle diagram of DEGs. (B) GO enrichment analysis of DEGs. (C) KEGG enrichment analysis of DEGs.

A

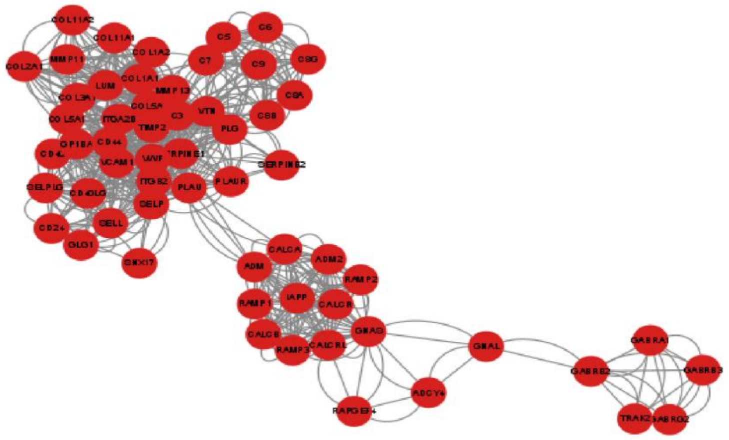

B

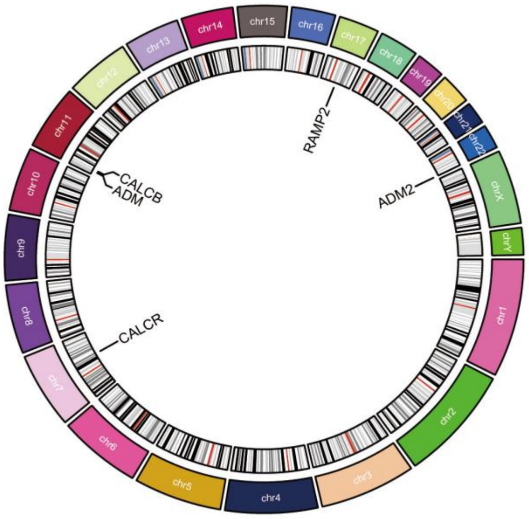

Figure S2. Identification and Function of Hub Genes. (A-B) Determine hub genes (DMNC and MCC). (C) The position of hub genes in chromosomes.

A

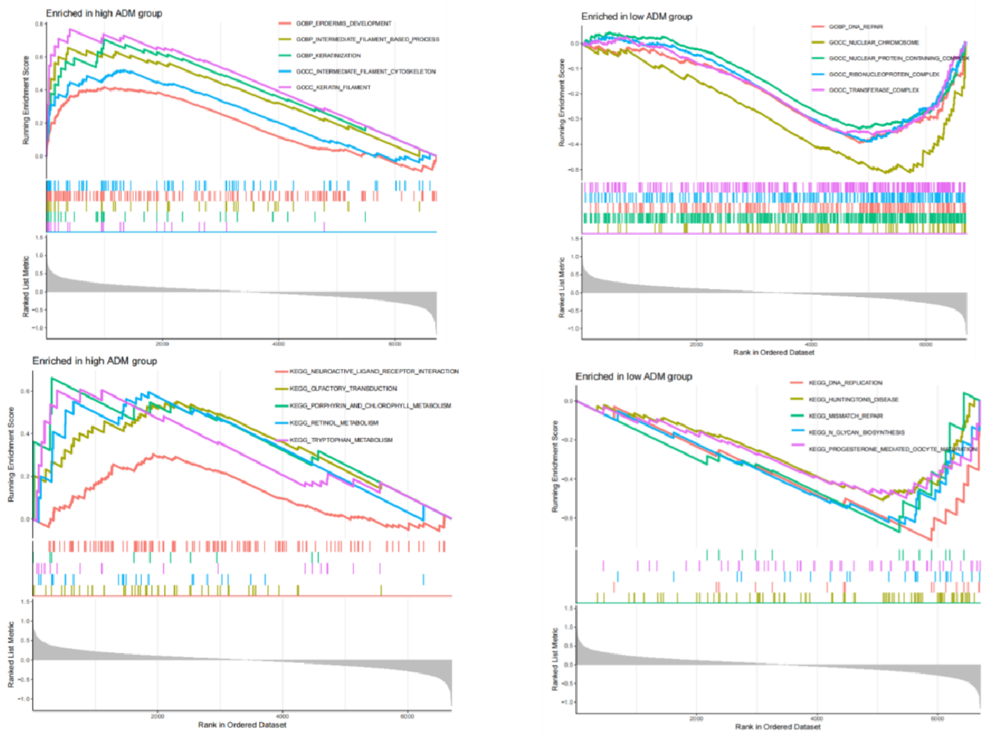

Enriched in high ADM group
GOBP_EPIDERMIS_DEVELOPMENT
GOBP_INTERMEDIATE_FILAMENT_BASED_PROCESS
GOBP_KERATINIZATION
GOCC_INTERMEDIATE_FILAMENT_CYTOSKELETON
GOCC_KERATIN_FILAMENT
Running Enrichment Score
Ranked List Metric
Enriched in low ADM group
GOBP_DNA_REPAIR
GOCC_NUCLEAR_CHROMOSOME
GOCC_NUCLEAR_PROTEIN_CONTAINING_CO
GOCC_RIBONUCLEOPROTEIN_COMPLEX
GOCC_TRANSFERASE_COMPLEX
Rank in Ordered Dataset
Enriched in high ADM group
KEGG_NEUROACTIVE_LIGAND_RECEPTOR_INTERACTION
KEGG_OLFACTORY_TRANSDUCTION
KEGG_PORPHYRIN_AND_CHLOROPHYLL_METABOLISM
KEGG_RETINOL_METABOLISM
KEGG_TRYPTOPHAN_METABOLISM
Enriched in low ADM group
KEGG_DNA_REPLICATION
KEGG_HUNTINGTONS_DISEASE
KEGG_MISMATCH_REPAIR
KEGG_N_GLYCAN_BIOSYNTHESIS
KEGG_PROGESTERONE_MEDIATED_OOCYTE_M

B

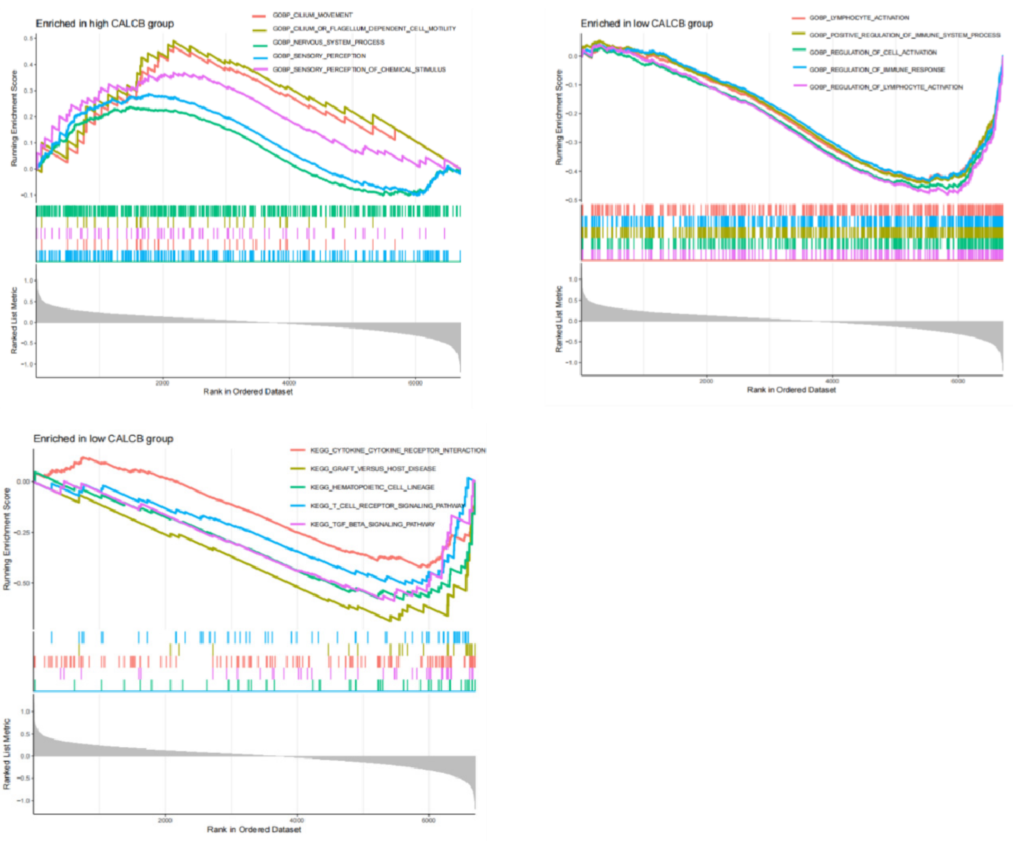

Enriched in high CALCB group
GOBP_CILIUM_MOVEMENT
GOBP_CILIUM_OR_FLAGELLUM_DEPENDENT_CELL_MOTILITY
GOBP_NERVOUS_SYSTEM_PROCESS
GOBP_SENSORY_PERCEPTION
GOBP_SENSORY_PERCEPTION_OF_CHEMICAL_STIMULUS
Running Enrichment Score
Ranked List Metric
Rank in Ordered Dataset
Enriched in low CALCB group
GOBP_LYMPHOCYTE_ACTIVATION
GOBP_POSITIVE_REGULATION_OF_IMMUNE_SYSTEM_PROCESS
GOBP_REGULATION_OF_CELL_ACTIVATION
GOBP_REGULATION_OF_IMMUNE_RESPONSE
GOBP_REGULATION_OF_LYMPHOCYTE_ACTIVATION
Enriched in low CALCB group
KEGG_CYTOKINE_CYTOKINE_RECEPTOR_INTERACTION
KEGG_GRAFT_VERSUS_HOST_DISEASE
KEGG_HEMATOPOIETIC_CELL_LINEAGE
KEGG_T_CELL_RECEPTOR_SIGNALING_PATHWAY
KEGG_TGF_BETA_SIGNALING_PATHWAY

C

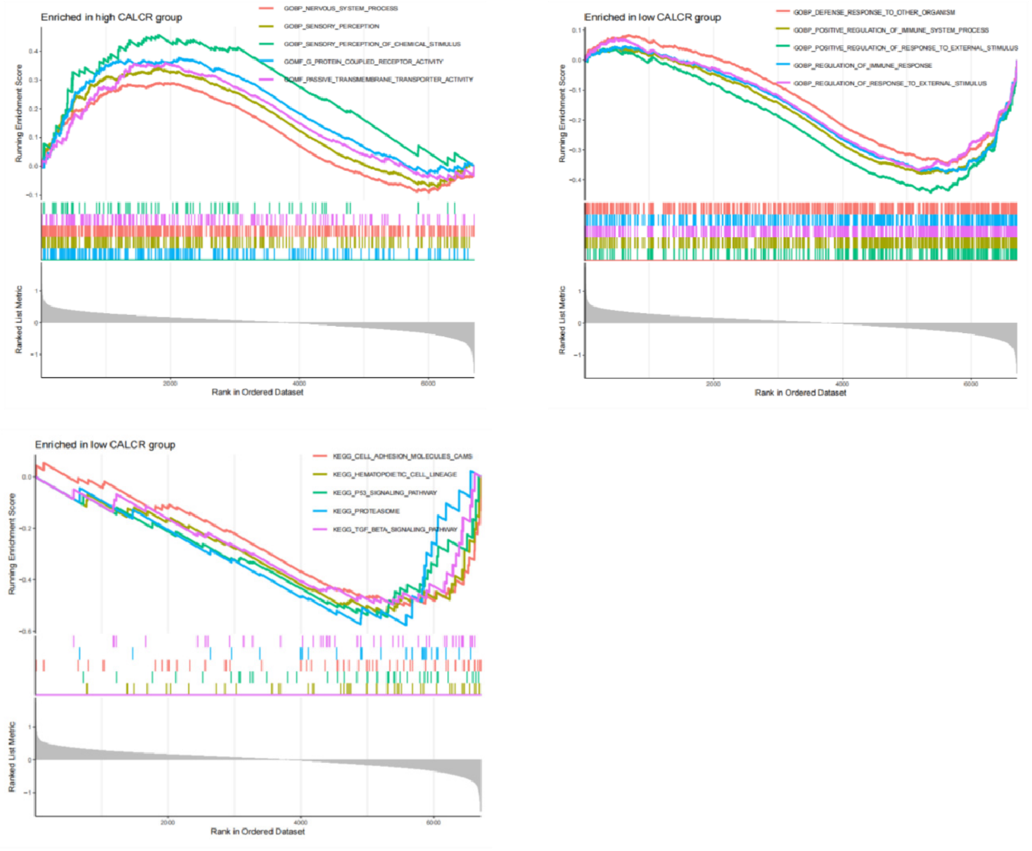

Figure S3. (A-C)Functional analysis of ADM, CALCB, and CALCR in sequence.
